# Applicability of Pharmacogenomically Guided Medication Treatment during Hospitalization of At-Risk Minority Patients

**DOI:** 10.3390/jpm11121343

**Published:** 2021-12-10

**Authors:** Loren Saulsberry, Keith Danahey, Merisa Middlestadt, Kevin J. O’Leary, Edith A. Nutescu, Thomas Chen, James C. Lee, Gregory W. Ruhnke, David George, Larry House, Xander M. R. van Wijk, Kiang-Teck J. Yeo, Anish Choksi, Seth W. Hartman, Randall W. Knoebel, Paula N. Friedman, Luke V. Rasmussen, Mark J. Ratain, Minoli A. Perera, David O. Meltzer, Peter H. O’Donnell

**Affiliations:** 1 Department of Public Health Sciences, The University of Chicago, Chicago, IL 60637, USA; 2 Center for Personalized Therapeutics, The University of Chicago, Chicago, IL 60637, USA; kdanahey@bsd.uchicago.edu (K.D.); mmiddlestadt@medicine.bsd.uchicago.edu (M.M.); dgeorge@bsd.uchicago.edu (D.G.); lhouse@medicine.bsd.uchicago.edu (L.H.); xvanwijk@bsd.uchicago.edu (X.M.R.v.W.); jyeo@bsd.uchicago.edu (K.-T.J.Y.); mratain@medicine.bsd.uchicago.edu (M.J.R.); podonnel@medicine.bsd.uchicago.edu (P.H.O.); 3 Center for Research Informatics, The University of Chicago, Chicago, IL 60637, USA; 4 Division of Hospital Medicine, Northwestern University Feinberg School of Medicine, Chicago, IL 60611, USA; Kevin.OLeary@nm.org; 5 Department of Pharmacy Systems, Outcomes and Policy, College of Pharmacy, University of Illinois Chicago, Chicago, IL 60612, USA; enutescu@uic.edu; 6 Center for Pharmacoepidemiology and Pharmacoeconomic Research, University of Illinois Chicago, Chicago, IL 60612, USA; 7 Section of Hospital Medicine, Department of Medicine, The University of Chicago, Chicago, IL 60637, USA; tchen2@medicine.bsd.uchicago.edu (T.C.); gruhnke@bsd.uchicago.edu (G.W.R.); dmeltzer@medicine.bsd.uchicago.edu (D.O.M.); 8 Department of Pharmacy Practice, University of Illinois Chicago, Chicago, IL 60612, USA; jamlee1@uic.edu; 9 Department of Pathology, The University of Chicago, Chicago, IL 60637, USA; 10 Advanced Technology Clinical Laboratory, The University of Chicago, Chicago, IL 60637, USA; 11 Department of Medicine, The University of Chicago, Chicago, IL 60637, USA; seth.hartman@uchospitals.edu (S.W.H.); randall.knoebel@uchospitals.edu (R.W.K.); 12 Committee on Clinical Pharmacology and Pharmacogenomics, The University of Chicago, Chicago, IL 60637, USA; 13 Department of Pharmacy, The University of Chicago, Chicago, IL 60637, USA; anish.choksi@uchospitals.edu; 14 Center for Pharmacogenomics, Department of Pharmacology, Northwestern University, Chicago, IL 60611, USA; paula.friedman@northwestern.edu (P.N.F.); minoli.perera@northwestern.edu (M.A.P.); 15 Department of Preventive Medicine, Northwestern University Feinberg School of Medicine, Chicago, IL 60611, USA; luke.rasmussen@northwestern.edu

**Keywords:** pharmacogenomics, implementation, minority populations

## Abstract

Known disparities exist in the availability of pharmacogenomic information for minority populations, amplifying uncertainty around clinical utility for these groups. We conducted a multi-site inpatient pharmacogenomic implementation program among self-identified African-Americans (AA; *n* = 135) with numerous rehospitalizations (*n* = 341) from 2017 to 2020 (NIH-funded ACCOuNT project/clinicaltrials.gov#NCT03225820). We evaluated the point-of-care availability of patient pharmacogenomic results to healthcare providers via an electronic clinical decision support tool. Among newly added medications during hospitalizations and at discharge, we examined the most frequently utilized medications with associated pharmacogenomic results. The population was predominantly female (61%) with a mean age of 53 years (range 19–86). On average, six medications were newly prescribed during each individual hospital admission. For 48% of all hospitalizations, clinical pharmacogenomic information was applicable to at least one newly prescribed medication. Most results indicated genomic favorability, although nearly 29% of newly prescribed medications indicated increased genomic caution (increase in toxicity risk/suboptimal response). More than one of every five medications prescribed to AA patients at hospital discharge were associated with cautionary pharmacogenomic results (most commonly pantoprazole/suboptimal antacid effect). Notably, high-risk pharmacogenomic results (genomic contraindication) were exceedingly rare. We conclude that the applicability of pharmacogenomic information during hospitalizations for vulnerable populations at-risk for experiencing health disparities is substantial and warrants continued prospective investigation.

## 1. Introduction

Prescription drugs lead to a number of serious, harmful side effects, including death. Genetic variation may contribute to inter-individual differences in adverse reactions and efficacy for many [1,2,3]. Investments in the field of pharmacogenomics, which identifies genetic variations influencing drug responses, have been made in hopes of capitalizing on the potential of precision medicine to enhance the safety and effectiveness of prescribing. At present, pharmacogenomics research across the translational cycle, which includes studies of scientific discovery, clinical trials, and implementation programs, frequently underrepresents minority populations. Despite this limitation, these studies provide the bulk of evidence fueling the rapid generation of new guidelines supporting the dissemination of emerging pharmacogenomic technologies and their implementation into mainstream clinical care.

The problem lies in that some genetic variations found in groups of European descent are rare in African American populations (and vice versa). Consequently, guidelines developed based primarily on genetic variation in groups of European descent may be significantly less useful in guiding care delivered to minorities. At worst, such guidelines dominantly based on European ancestry populations may be incorrect and could potentially introduce harm with inappropriate pharmacogenomic advice (e.g., false assurances a medication will work or failing to indicate appropriate risk for adverse events). Lack of representation of diverse populations in pharmacogenomics research has exacerbated uncertainty around its clinical utility for minority patients and threatens their equitable realization of the potential health benefits. Amidst concerns that this underrepresentation may contribute to well-documented health disparities throughout the healthcare system [4], pharmacogenomics is increasingly being considered in clinical practice and is being advocated for greater use as a step toward precision medicine [5].

Possibly related to the dearth of studies incorporating diverse populations, limited research to date has evaluated the potential impact of pharmacogenomics on health disparities [6]. In this study, we evaluated the first multi-site pharmacogenomic implementation program, to our knowledge, that focuses on African American adults treated within the hospital setting. We specifically examined the availability of patient-specific pharmacogenomic information at the point-of-care via an institutional clinical decision support tool to inpatient providers caring for frequently hospitalized patients. We hypothesized that evidence-based pharmacogenomic information would be relevant to the medications used in this setting for hospitalized African American patients.

## 2. Materials and Methods

### 2.1. ACCOuNT Consortium Translational Project

The ACCOuNT (African American Cardiovascular Pharmacogenomics Consortium) Translational Project aimed to evaluate the clinical translation of pharmacogenomics to minority patients historically excluded from genomic studies. The ACCOuNT clinical trial was a multi-site study including The University of Chicago, the University of Illinois Chicago (UIC), and Northwestern University. The overall study conceptualization and schema have been previously described [7]. Patients who were hospitalized at any of these three institutions were approached for enrollment and collection of a blood sample for broad pharmacogenomic genotyping. Then, results were made available for subsequent hospitalizations to enrolled treating providers (physicians, advance practice providers, and pharmacists) via an electronic decision support tool, and prescribing during the hospitalization and at discharge were evaluated.

### 2.2. Study Population and Setting

The period of our analysis extended from 12 July 2017 to 18 June 2020, during which time self-identified African-American adults (18 years or older) who were English speakers were recruited to participate during an admission into the hospital. Following consent and study enrollment, patients received preemptive genetic testing using a broad custom pharmacogenomic panel. Genotyping was completed by the Advanced Technology Clinical Laboratory at the University of Chicago and was performed according to the previously described methods in a CLIA-certified and CAP-accredited laboratory [8,9,10]. Treating providers representing the care teams during inpatient stays were also recruited and enrolled into the study so that prescribing could be analyzed. This included hospitalist physicians (MDs), physician assistants (PAs), advanced practice nurses (APNs), and pharmacists (PharmDs). These providers were given access to the GPS and patient-specific pharmacogenomic results. As described previously at the point-of-care, clinical decision support within the GPS was provided with clinical annotations that communicate drug risk (low genomic risk vs. cautionary vs. high genomic risk) based on patient-specific test results [8,11,12]. Providers were notified of available pharmacogenomic results.

### 2.3. Applicability of Pharmacogenomic Results during Hospitalization

Evaluable hospital encounters included all rehospitalizations to qualifying inpatient services at the enrolling institution after completion of that patient’s preemptive genotyping. Qualifying inpatient services at the University of Chicago included the advanced practice service (APS), also known as the short-stay unit (SSU), as well as the Hospitalist services excluding the Hospitalist teaching services. Northwestern’s evaluable services were the Hospitalist services including the teaching services. UIC’s internal medicine service, excluding their teaching services, were considered qualifying inpatient services.

Medications prescribed during each evaluable readmission were recorded manually by research coordinators at each of the enrolling institutions through extraction from the electronic medical records. Admission (“baseline”) medication lists were collected, annotated with all medications added during the hospitalization, and then compared with final discharge medication lists. To ensure data quality, the medication list data for approximately 10% of the subjects were verified by a second reviewer (at an independent time point). All medications, including PRN (as needed) medications, were counted if they were prescribed in the study regardless of whether they were actually administered during the hospitalization (e.g., for PRN medications) or taken (after discharge). Medications were analyzed in this study according to reported prescriptions, or the number of orders, for distinct drugs. We also performed Pearson correlation analyses to better understand how patient characteristics were associated with medications and rehospitalizations.

We analyzed the availability of pharmacogenomic results via the GPS by medication type during these evaluable rehospitalizations. We categorized prescribing events into one of two “types” during the hospital stay: newly prescribed medications and discharge medications. Newly prescribed medications represented prescriptions that were added during the hospital encounter. Discharge medications were defined as medications prescribed to patients when they left the hospital at the conclusion of their inpatient stay. For each patient hospital admission, newly added medications also prescribed at discharge were only considered newly prescribed medications in our evaluation.

Within each of these categories, we tabulated and characterized the most frequently prescribed medications having annotated pharmacogenomic results available via GPS. These evaluations assessed the risk level of patient pharmacogenomic results by “traffic light” color in the GPS. Green lights indicated low-risk (genomically favorable) pharmacogenomic results. Yellow lights indicated cautionary (genomically discordant) results. Red lights represented high-risk (genomically contraindicated) results. Each light-categorized result was also accompanied by a clinical decision support summary containing clinically relevant information for each drug–genomic pair for prescribing guidance.

The primary endpoints were the percentage of medications prescribed to frequently rehospitalized African Americans with cautionary or high-risk pharmacogenomic information, both during inpatient stays and at discharge.

### 2.4. Analysis of Frequency of Rehospitalization and Length of Hospital Stay Based on Pharmacogenomic Medication Risk

As an exploratory endpoint of this study, we sought to understand whether pharmacogenomic discordance (yellow or red light results) for prescribed medications correlated with either of two undesirable outcomes for this frequently hospitalized population: annual frequency of rehospitalization and/or length of stay.

Since patients were enrolled at different times during the overall study period, we first normalized the length of study participation for each patient to an annualized number of observation days using the first date of first evaluable hospital readmission and the study end date (or date of death if patient expired prior to the end of the study period, whichever came first). Patients whose length of observation period was less than 90 days were excluded from the rehospitalization frequency and length of stay analyses because the observation period was too short to draw reliable conclusions. Frequency of hospitalization was expressed as the number of hospitalizations (at the enrolling institution) per year (i.e., calculated by counting the number of total rehospitalizations divided by the observation period, and expressed as an annualized frequency). Length of stay (number of inpatient hospital days) was calculated for every evaluable hospital readmission per patient.

We examined whether there was a relationship between length of hospital stay or frequency of rehospitalization and the average number of cautionary pharmacogenomic results per patient-admission, using Pearson and Spearman rank correlation, with the level of statistical significance set at *p* < 0.05 for this exploratory analysis.

## 3. Results

### 3.1. Patient Characteristics

Table 1 shows the baseline characteristics of our final study population, which consisted of 135 African American (AA) patients that were readmitted to the hospital over the study period. Patients ranged in age from 19 years to 86 years, with an average age of 53 years. Most patients (79%) in our population were 40 years or older. The majority (61%) of AA patients were female, and 84% percent of study participants reported an educational attainment of some college or less.

AA patients in our study population had multiple comorbidities, or co-existing health problems, which are the top drivers of morbidity and mortality for the African American population [13]. Table 1 displays the most prevalent reported health problems in our study population. On average, at the time of study enrollment, AA patients had some combination of seven of these most prevalent health problems. The most frequent health problems related to respiratory disorders, hypertension, heart disease, neurological findings (e.g., pain, numbness, etc.), and diabetes. AA patients in our study population were also exposed to prescriptions for several medications (polypharmacy) at the time of study enrollment. The majority (60%) of hospitalized patients had 10 or more drug prescriptions at baseline (at the time of study enrollment) with an average of 12 drug prescriptions per patient (range: 1–28).

We found that polypharmacy was significantly correlated with having a higher number of comorbidities (r = 0.50, *p* < 0.001), but it was not correlated with age, gender, or education level.

### 3.2. A Population with Frequent Rehospitalization Risk

The average observation period per patient was 1.35 years (range: 0.008–2.94 years) or about 493 days (range: 3–1072 days). The average number of rehospitalizations per patient was 3 (range: 1–15). After considering the per patient observation periods, this translates into an average rate of rehospitalization for the patients in our cohort of four admissions per patient-year (range: 1–121). Overall, the study population experienced 341 rehospitalizations from 12 July 2017 to 18 June 2020, and the mean length of each hospital stay was 7 days with a range of 1 to 33 days (Table 2). We found that an increased number of rehospitalizations was significantly correlated with lower patient age (r = −0.20, *p* < 0.05) but was not correlated with gender, education, or number of comorbidities.

### 3.3. Inpatient Medication Prescribing Was Commonly Associated with Applicable Pharmacogenomic Results

Among patients rehospitalized at least once, one to 17 newly prescribed medications were prescribed during a rehospitalization with an average of six newly prescribed medications per readmission. For these newly prescribed medications, the average number of associated pharmacogenomic results available in the GPS for a single patient was two (range: one to six pharmacogenomic results). For medication prescriptions at discharge, the average number of associated pharmacogenomic results available in the GPS for a single patient was three (range: one to nine pharmacogenomic results).

**Figure 1:**
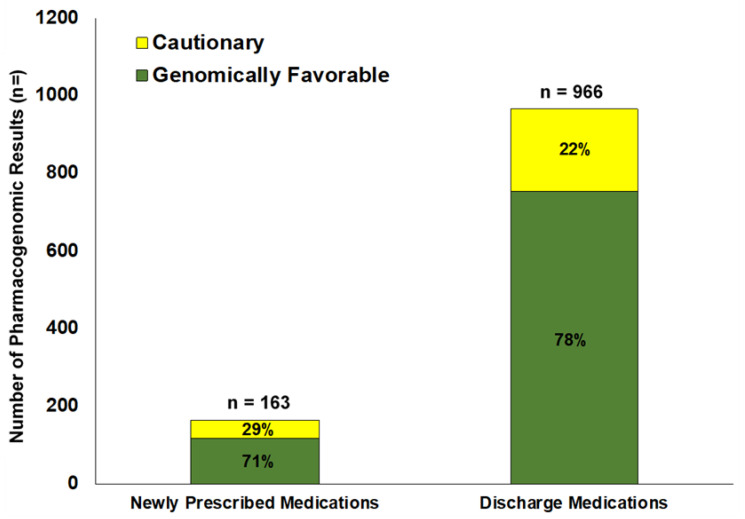
Pharmacogenomic results available during hospitalizations of African American patients by medication type and genomic risk level. Results are shown for *n* = 135 African American patients with readmissions to the hospital from 12 July 2017 to 18 June 2020. Pharmacogenomic results available via the GPS at the point-of-care included test results that were genomically favorable (green light; compatible), cautionary (yellow light; potentially increased genomic risk), and genomically unfavorable (red light; genomic contraindication). Only two high-risk pharmacogenomic results (red lights) were observed during the study period (both as discharge medications); these are not displayed in Figure 1 as they constituted <1% of all results. Pharmacogenomic results were accompanied by clinical summaries to guide drug treatment.

Pharmacogenomic results were available to hospital providers via GPS for at least one medication during 48% of rehospitalizations. A total of 163 newly prescribed medications involving 22 distinct drugs with evidence-based pharmacogenomic information in the GPS were prescribed across all of the inpatient readmissions. Separately, AA patients were discharged on a total of 966 prescribed medications over the course of the study period (Figure 1). Figure 1 shows the varying risk levels (low risk or cautionary) of the pharmacogenomic results available in the GPS for prescription medications, as recorded by medication category (newly prescribed during the readmission or prescribed at discharge). Most of the pharmacogenomic results available for medications prescribed to AA patients over the course of their hospitalizations were for low-risk, genomically favorable medications. However, nearly one out of every three newly prescribed medications (29%) and approximately one of every five discharge medications (22%) were associated with actionable pharmacogenomic results, indicating a cautionary risk (genomic discordance; increased risk of toxicity or increased chance of suboptimal response). No actionable patient pharmacogenomic results for newly prescribed inpatient medications indicated high risk for unfavorable drug response (no red lights). Among all discharge medications, two high risk pharmacogenomic results for omeprazole were identified, constituting <1% of all pharmacogenomic results available for discharge medications.

The medications newly prescribed during rehospitalizations treated a range of health conditions, and there was significant variability in the strength of association with cautionary pharmacogenomic information (Table 3). Drugs for the treatment of gastroesophageal disease, asthma, hypertension, hypercholesterolemia, and heart failure were most commonly associated with cautionary pharmacogenomic signals.

### 3.4. Cautionary Pharmacogenomic Results for Medications Newly Prescribed during Rehospitalization Were Associated with Increased Length of Hospital Stay

**Figure 2:**
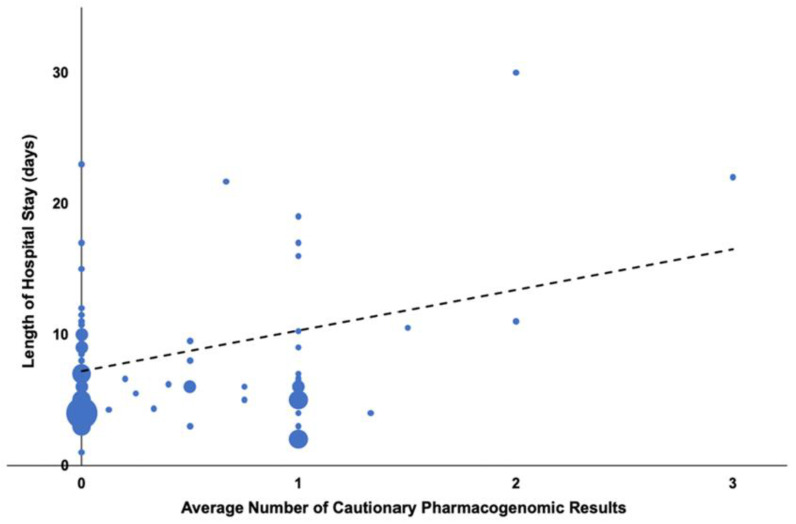
Cautionary pharmacogenomic results available for newly prescribed medications during hospitalizations. There was a positive relationship between the per-patient average number of cautionary pharmacogenomic results associated with newly prescribed medications during hospitalizations and length of hospital stay; however, the correlation was modest, r (71) = 0.33, *p* < 0.01 Pearson’s r. Since this correlation may have been driven by a small number of patients with a higher number of cautionary pharmacogenomic results, we repeated the analysis using Spearman’s rank correlation test, which may provide a more robust assessment in the presence of outliers, and we observed a small non-significant correlation (ρ = 0.09, *p* = 0.44).

We found that patients, on average, received approximately one new medication per admission that was associated with cautionary pharmacogenomic information. Evaluation of these cautionary pharmacogenomic results available for newly prescribed medications during rehospitalizations showed a positive correlation between a higher average number of cautionary pharmacogenomic results and increased length of hospital stay (Figure 2, r = 0.33, *p* < 0.01 Pearson’s r). Analysis of the Figure 2 data showed that this correlation may have been driven by a small number of patients with a higher number of cautionary pharmacogenomic results. Consequently, we repeated the analysis using Spearman’s rank correlation test, which may provide a more robust assessment in the presence of outliers, and we found a small non-significant correlation (ρ = 0.09, *p* = 0.44). This suggests that a patient may need to be prescribed multiple genomically discordant medications or potentially one or more high-risk (“red light”) medications (which were almost never observed in this population) in order to drive increased length of hospital stay. In a separate analysis, we found no association between cautionary pharmacogenomic results for newly prescribed medications and frequency of rehospitalization.

## 4. Discussion

In this study, we evaluated the availability of evidence-based pharmacogenomic information to providers at the point-of-care for African American patients with frequent hospitalizations. For almost half (48%) of all hospitalizations, clinical pharmacogenomic information was available to providers during their inpatient stay for at least one newly prescribed medication. Notably, more than one in five of both newly prescribed and discharge medications for patient rehospitalizations were associated with cautionary pharmacogenomic risk. The most frequent newly prescribed medications associated with cautionary pharmacogenomic results are commonly used to treat chronic conditions prevalent within our hospitalized African American patient cohort. Nevertheless, high-risk pharmacogenomic results (genomic contraindication) were rare and only observed for discharge medications. Finally, we found a positive association between the average number of cautionary pharmacogenomic results and increased length of hospital stay.

Our study participants had an average of seven chronic comorbidities, and, during the study period, they averaged four rehospitalizations/year. We showed that for the evaluated rehospitalizations, 29% of newly prescribed medications and 22% of discharge medications were associated with pharmacogenomic results, indicating genomic discordance or cautionary risk. Considered together, our results and prior literature suggest that these vulnerable patients are at increased cumulative risk for potential adverse drug responses (ADRs) compared to other groups [4,5]. Since germline pharmacogenomic testing is relevant to drug treatment across multiple therapeutic areas and retains relevancy over the course of a patient’s lifetime, the findings of this study indicate that the availability of pharmacogenomic information may facilitate a reduction of cumulative risk for healthcare disparities risk that African Americans typically experience.

Our results revealed that of the more frequent newly prescribed medications within our hospitalized African American cohort, those treating gastroesophageal disease, asthma, hypercholesterolemia, hypertension, and heart failure were associated with cautionary pharmacogenomic results. These therapeutic areas involving medications with clinically relevant pharmacogenomic information may denote clinical areas where targeted genotyping could occur in resource-constrained clinical environments to best optimize prescribing practices for minority patients. Ambulatory care-sensitive conditions (ACSCs), where proper treatment and management is believed to prevent avoidable hospital admissions, include chronic conditions such as asthma, hypertension, and diabetes—conditions prevalent both in our study’s patient cohort and generally within the African American population [14,15,16]. Multiple studies have examined patterns of hospitalization due to ACSCs by race/ethnicity, and these have reported higher rates of hospitalizations for African American compared to Whites [17,18,19,20,21,22,23,24,25]. Although African Americans have been shown to be at greater risk for mortality due to ADRs [26], it is unclear how race might act as a predictor for ADRs across different healthcare settings, especially when the context of the social determinants of health and access barriers (e.g., health insurance) are considered.

Considering the rate of high-risk pharmacogenomic results (red lights; genomic contraindication) available across race/ethnicity for patients participating in previous studies within the outpatient setting [8], the extreme rarity of these high-risk results in the current study was surprising. Despite its enormous potential to positively impact medication treatment and avoid ADRs, pharmacogenomics has evolved based on studies that persistently underrepresent populations of diverse genetic ancestry [27,28]. Consequently, the limited availability of high-risk pharmacogenomic results within this study could indicate that the current evidence base may not incorporate the most relevant genetic variants for assessing genomic risk among African American patient populations. Future research exploring differences in the prevalence of pharmacogenomic risk alleles between diverse patient populations is warranted. Socioeconomic inequalities contributing to reduced healthcare access have been demonstrated to impact numerous health outcomes including hospitalizations for chronic conditions [15], yet the contributions of such broader socioeconomic circumstances to the assessment of potential variation in pharmacogenomic risk have yet to be well-characterized across health systems and clinical settings.

Our finding of a potential relationship between the average number of cautionary pharmacogenomic results and increased length of hospital stay is interesting and could lead to several interpretations that merit further investigation. First, it could be possible that a patient may need to be prescribed multiple genomically discordant medications, or potentially one or more high-risk medications (e.g., red lights—which were almost never observed in our exclusively African American population), in order to drive increased length of hospital stay. Alternatively, because this relationship was relatively weak and because we did not find a similar correlation between the number of cautionary pharmacogenomic results and frequency of hospitalization, it may underscore instead the idea that the pharmacogenomic information collected and employed in clinical settings which overwhelmingly comes from populations of European ancestry may be less relevant for minority patients. Either way, while no causal inferences can currently be made, future research should evaluate whether longer hospital stay simply increases the risk of exposure to more medications, or whether medications associated with cautionary pharmacogenomic results place patients at greater risk or cause ADRs that then require lengthier hospital stays.

This study had some limitations. As our study sample included only self-identified African Americans, all study findings may not be generalizable to all racial/ethnic patient populations. However, this may actually be perceived as a strength of our study that we exclusively examined an underrepresented patient population. Nevertheless, our study design incorporating multiple institutional sites strengthens the likelihood that our results may be generalizable to African American populations across diverse medical care settings. Secondly, this study did not evaluate actual drug administrations nor did we investigate outpatient consequences beyond the hospitalization. Finally, we did not look at actual ADRs or other health outcomes that may be significant. However, these important considerations are being collected within prospective follow-up studies.

Prior studies examining pharmacogenomic implementation among diverse populations have failed to establish any consensus around the clinical utility of pharmacogenomics in minority patient populations. While efforts, such as the Precision Medicine Initiative [29] and All of Us [30], are aimed at increasing the availability of genetic information from diverse populations to catalyze genomic innovations, studying the relevance and impact of pharmacogenomic implementation for minority patients across clinical settings is critical. Our current findings add to this emerging body of evidence by showing that inpatient care setting presents unique opportunities to intervene in improving minority patient care. In particular, an opportunity exists to facilitate provider access to pharmacogenomic information, which may avoid potential adverse drug responses and optimize medication treatment for minority patients. Future research expanding upon these findings will help to understand the degree to which medically underserved populations at high risk for rehospitalization and polypharmacy may more equitably benefit from genomic innovations such as the routine availability of pharmacogenomics.

## Figures and Tables

**Table 1:** Baseline characteristics of hospitalized African American patients. Percentages (%) may not sum to 100 due to rounding. Reflects UChicago, Northwestern, University of Illinois at Chicago. Reflects total number of patients with rehospitalizations within the time range (12 July 2017–18 June 2020).

	Number of Patients with Hospitalizations (*n* = 135)
Characteristic	
Age in years [mean (range)]	53 (19–86)
18–25 years [n (%)]	6 (4)
26–39 years [n (%)]	23 (17)
40–50 years [n (%)]	35 (26)
51–64 years [n (%)]	36 (27)
65+ years [n (%)]	35 (26)
Gender [n (%)]	
Female	83 (61)
Male	52 (39)
Education [n (%)]	
HS or less	60 (44)
Some college	54 (40)
College graduate	16 (12)
Advanced degree	5 (4)
Top Health Problems (Rank)	
1	Respiratory Disorders
2	Hypertension
3	Heart Disease
4	Neurological Findings (Pain, Numbness, etc.)
5	Diabetes
6	Arthropathy
7	Cholesterol
8	Gastrointestinal Tract Disorder
9	Psychiatric Illness (Anxiety/Depression)
10	Kidney Disease
Number of Comorbidities [n (%)]	
mean # of health problems (range)	7 (1–19)
1–3 health problems	33 (24)
4–6 health problems	34 (25)
7–9 health problems	33 (24)
10+ health problems	32 (24)
Most Prevalent Medications (Rank)	
1	Salbutamol/Albuterol
2	Acetaminophen
3	Aspirin
4	Atorvastatin
5	Gabapentin
6	Amlodipine
7	Furosemide
8	Insulin Glargine
9	Lisinopril
10	Metoprolol
Polypharmacy [n (%)]	
mean # of prescription drugs (range)	12 (1–28)
1–3 prescription drugs	8 (6)
4–6 prescription drugs	14 (10)
7–9 prescription drugs	32 (24)
10 or more prescription drugs	81 (60)
10–12	23 (17)
13–15	23 (17)
16–20	23 (17)
21–28	12 (9)

**Table 2:** Frequency of patient hospitalizations. Includes all *n* = 135 African American patients with at least one readmission to the hospital during the three-year study period.

Characteristics	
Total number of hospitalizations (after enrollment) [n]	341
Hospitalizations per patient (after enrollment) [n (%)]	
mean (range)	3 (1–15)
1	69 (51)
2	22 (16)
3–4	24 (18)
5+	20 (15)
Length of hospital stay (days) [mean (range)]	7 (1–33)

**Table 3:** Pharmacogenomic results available for newly prescribed medications during hospitalizations. All pharmacogenomic results that were available via the GPS at the point-of-care for newly prescribed medications during the study are shown. Results were categorized as genomically favorable (green light) vs cautionary genomic risk (yellow light) based on each individual patient’s genetic test results. Providers accessing individual results received detailed clinical decision support summaries to guide/inform prescribing.

Newly Prescribed Medications (Name)	Gene(s)	Pharmacogenomic Relevance	Total Unique Pharmacogenomic Results (n = )	Genomically Favorable [n (%)]	Cautionary [n (%)]
PANTOPRAZOLE	*CYP2C19*	Suboptimal antacid response	39	13 (33)	26 (67)
TRAMADOL	*CYP2D6*	Suboptimal response (lack of analgesia) or exaggerated response (increased risk of toxicity)	22	20 (91)	2 (9)
HYDRALAZINE	*NOS3*	Suboptimal heart failure response	16	10 (63)	6 (38)
MORPHINE	*OPRM1*	Suboptimal response (lack of analgesia)	15	14 (93)	1 (7)
OXYCODONE	*CYP2D6*	Suboptimal response (lack of analgesia) or exaggerated response (increased risk of toxicity)	14	13 (93)	1 (7)
BUDESONIDE	*GLCCl1*	Suboptimal asthma response	13	13 (100)	0 (0)
FLUTICASONE PROPIONATE	*GLCCl1*	Suboptimal asthma response	13	13 (100)	0 (0)
CODEINE	*CYP2D6*	Suboptimal response (lack of analgesia) or exaggerated response (increased risk of toxicity)	9	9 (100)	0 (0)
ASPIRIN	*LTC4S*	Increased risk of urticaria	7	6 (86)	1 (14)
IBUPROFEN	*CYP2C9*	Increased risk of gastrointestinal bleeding	7	7 (100)	0 (0)
METOPROLOL	*CYP2D6*; *ADRB1*; *GRK4*	Suboptimal response (blood pressure/ejection fraction) or increased risk of bradycardia	7	7 (100)	0 (0)
CARVEDILOL	*ADRB1*; *ADRB2*	Suboptimal ejection fraction response	6	1 (17)	5 (83)
AMLODIPINE	*CACNA1C; CYP3A4*	Suboptimal blood pressure lowering response	4	1 (25)	3 (75)
CLOPIDOGREL	*CYP2C19*	Increased risk of cardiovascular events (heart attack, stroke, and/or mortality)	4	2 (50)	2 (50)
DULOXETINE	*COMT*	Suboptimal response (treatment of depression)	4	2 (50)	2 (50)
ISOSORBIDE DINITRATE	*NOS3*	Suboptimal heart failure response	4	2 (50)	2 (50)
ATORVASTATIN	*SLCO1B1; KIF6; GNB3*	Elevated risk of statin-induced adverse events (myopathy); suboptimal cholesterol-lowering effect	3	3 (100)	0 (0)
HYDROCHLOROTHIAZIDE	*ADD1*	Suboptimal blood pressure lowering response	3	1 (33)	2 (67)
ESOMEPRAZOLE	*CYP2C19*	Suboptimal antacid response	2	2 (100)	0 (0)
MONTELUKAST	*ABCC1*	Suboptimal asthma response	2	0 (0)	2 (100)
METFORMIN	*SLC22A1*	Suboptimal reduction in hemoglobin A1c (blood sugar)	1	1 (100)	0 (0)
NIFEDIPINE	*CACNA1C*	Suboptimal blood pressure lowering response	1	1 (100)	0 (0)
SIMVASTATIN	*SLCO1B1*	Elevated risk of statin-induced adverse events (myopathy)	1	0 (0)	1 (100)
TRIAMCINOLONE	*GLCCl1*	Suboptimal asthma response	1	1 (100)	0 (0)

## Data Availability

The data underlying this article will be shared on reasonable request to the corresponding author.
